# A Functional Synonymous Variant in *PDGFRA* Is Associated with Better Survival in Acral Melanoma

**DOI:** 10.7150/jca.43010

**Published:** 2020-03-04

**Authors:** Jie Dai, Lu Yang, Tianxiao Xu, Lu Si, Chuanliang Cui, Xinan Sheng, Zhihong Chi, Lili Mao, Bin Lian, Bixia Tang, Xue Bai, Li Zhou, Siming Li, Xuan Wang, Xieqiao Yan, Yan Kong, Jun Guo

**Affiliations:** 1 Key Laboratory of Carcinogenesis and Translational Research (Ministry of Education/Beijing), Department of Renal Cancer and Melanoma, Peking University Cancer Hospital and Institute, Beijing 100142, China; 2 Department of Radiology, Peking University Shougang Hospital, Beijing 100144, China

**Keywords:** acral melanoma, *PDGFRA*, genetic variation, gene expression regulation, survival

## Abstract

**Purpose**: Polymorphisms of genes in the platelet-derived growth factor (PDGF) signaling pathway have been found to predict cutaneous melanoma (CM) survival, but their clinical effects in acral melanoma (AM) patients have not been explored. The aim of this study was to characterize the functional effect of the tag single-nucleotide polymorphism (SNP) rs2228230:C>T and assess its association with clinical outcomes in AM patients.

**Methods**: The effect of rs2228230:C>T on mRNA structures and codon usage values were evaluated using *in silico* analyses. PDGF receptor alpha (*PDGFRA*) expression vectors with the rs2228230:C or rs2228230:T allele were constructed to evaluate the expression and signaling activity of *PDGFRA*. The expression of *PDGFRA* in AM samples was measured using *in situ* RNAscope hybridization and immunohistochemical staining. The association of the rs2228230 genotype with survival was analyzed in two independent AM cohorts.

**Results**: *In silico* analyses indicated that the rs2228230:T allele increases the minimum free energy and reduces synonymous codon usage. The rs2228230:T allele decreased the expression of *PDGFRA* by reducing the stability of its mRNA and protein as well as the signaling activity of the MAPK and PI3K/AKT pathways. *PDGFRA* mRNA and protein expression was significantly reduced in AM tissues with the rs2228230:T allele. The progression-free survival and overall survival of AM patients with the rs2228230:T allele were significantly longer than those of patients with the CC genotype.

**Conclusion**: Our study indicated that rs2228230:T can reduce the expression of *PDGFRA* and downstream signaling activity and is associated with better survival in AM patients.

## Introduction

Platelet-derived growth factor (PDGF) receptor alpha (*PDGFRA*) encodes a cell surface tyrosine kinase receptor, which binds to three forms of PDGFs and forms a homodimer or heterodimer, thereby mediating many biological processes, including organ development, wound healing, angiogenesis, cell proliferation, and differentiation 1,2. PDGFRA plays a role in tumor progression, and mutations in *PDGFRA* have been associated with idiopathic hypereosinophilic syndrome 3, gastrointestinal stromal tumors 4, and several other cancers 5. We and others have demonstrated that mutations, increased copy numbers, and overexpression of *PDGFRA* occur in melanoma patients, and that functional mutations of *PDGFRA* increase MAPK and PI3K/AKT pathway activation, which can be inhibited by several tyrosine kinase inhibitors such as imatinib, crenolanib, and sunitinib 6-8.

Besides functional mutations, polymorphisms can also influence susceptibility to disease and response to anticancer drug treatment. A genome-wide association study (GWAS) in Australian and European populations found that the single-nucleotide polymorphism (SNP) rs3219090 within *PARP1* is a melanoma susceptibility locus 9, and subsequent studies demonstrated that the rs3219090 risk allele is correlated with higher expression levels of *PARP1* mRNA but longer melanoma-specific survival 10,11. The polymorphism rs333 (*CCR5Δ32*) within *CCR5* results in premature translation termination, and a study on a large melanoma cohort found that this polymorphism was associated with decreased overall survival (OS) in stage IV melanoma patients treated with immunotherapy 12. The genetic variants of members of the PDGF signaling pathway have been demonstrated to predict cutaneous melanoma (CM) survival in patients in the United States 13. Unlike in Caucasians, in whom the most prevalent melanoma subtype is non-acral CM, the major subtype in Asians is acral melanoma (AM), which accounts for 42.8% of melanoma cases in China 14. However, the clinical relevance of *PDGFRA* polymorphisms in AM patients has not been explored yet.

rs2228230:C>T is the most common and only tag SNP located within the coding sequence of *PDGFRA* (Figure S1) 15. The tag SNP was mapped to the ATP-binding site and polypeptide substrate-binding site in a conserved protein kinase (PTKc) domain; furthermore, it lies within the exonic splicing enhancer/exonic splicing silencer (ESE/ESS)-binding sites 16, which may disrupt mRNA splicing and affect protein function. The minor T allele of rs2228230 in infants was reported to be associated with a reduced risk of obstructive heart defects after alcohol exposure during the periconceptional period 17. Meanwhile, the genotype of rs2228230 was found to be significantly associated with corneal astigmatism according to a meta-analysis of five GWASs of 8,513 Asian individuals 18. The rs2228230:T allele has also been demonstrated to be associated with worse disease-free survival in renal cell carcinoma in a Spanish population 19. Therefore, in this study, we investigated the functional effect of rs2228230 through *in silico* and *in vitro* studies as well as the association of the rs2228230 genotype with *PDGFRA* expression and clinical outcomes of AM patients in two independent cohorts.

## Materials and Methods

### Study participants

In this retrospective exploratory study, data from patients diagnosed with melanoma who received standard treatment from Peking University Cancer Hospital and Institute between January 2010 and August 2017 were reviewed. A total of 546 AM patients were enrolled in the discovery cohort, and 256 AM patients were enrolled in the replication cohort. A group of 240 CM patients were enrolled in an independent cohort. The last follow-up was carried out in March 2019; the median follow-up times were 33.2 months (range, 0.2-103.8 months), 25.9 months (range, 1.6-94.7 months), and 31.0 months (range, 0.6-91.5 months). All procedures were approved by the Ethics Committee of Peking University Cancer Hospital and Institute and were conducted in adherence to the principles of the Declaration of Helsinki. Informed consent for the use of specimens was obtained from all participants.

### Cell culture

The HEK293 cell line was obtained from the Cell Bank of the Chinese Academy of Sciences and cultured in Dulbecco's modified Eagle medium (DMEM) supplemented with 10% fetal bovine serum, 100 U/mL penicillin, 100 mg/mL streptomycin, and 2 mM GlutaMAX at 37 °C in a humidified incubator with 5% CO_2_. All reagents used for cell culture were purchased from Gibco (Grand Island, NY, USA). The cell line was authenticated using short tandem repeat (STR) profiling and was confirmed to be negative for mycoplasma.

### DNA extraction and genotyping

Genomic DNA was extracted from formalin-fixed, paraffin-embedded (FFPE) tissue sections using the QIAamp DNA FFPE Tissue Kit (Qiagen, Hilden, Germany). Polymerase chain reaction (PCR)-amplified products were subjected to Sanger sequencing to determine the genotypes of rs2228230 according to our previous report 6. Chromatogram results were further analyzed by BLAST and manual review.

### RNA extraction and quantitative reverse transcription PCR

Total RNA was extracted using the RNeasy Mini Kit (Qiagen). Two micrograms of RNA was reverse transcribed into cDNA using the ReverTra Ace qPCR RT Kit (Toyobo, Osaka, Japan). Quantitative reverse transcription PCR (qRT-PCR) was performed using GoTaq® qPCR Master Mix (Promega, Madison, WI, USA) on an ABI 7500 FAST Real-Time PCR System (Applied Biosystems, Foster City, CA, USA). *GAPDH* was used as an internal control, and the relative quantitative levels of *PDGFRA* expression were calculated by the 2^-ΔΔCt^ method.

### RNA *in situ* hybridization of *PDGFRA*

*PDGFRA* mRNA in FFPE melanoma samples was measured manually using the RNAscope assay (Advanced Cell Diagnostics, ACD, Hayward, CA, USA). After deparaffinization and incubation with pretreatment reagents, the slides were hybridized with Hs-PDGFRA-probes (ACD Probe: 604481) in a HybEZ oven (ACD) at 40 °C for 2 h. Hybridization signals were amplified and detected using the RNAscope 2.5 HD Detection Kit (Red; ACD, 322360). After image capture, digital *in situ*-hybridized slides were analyzed by Fiji (ImageJ) software. Positive signals appeared as red dots. Human peptidylprolyl isomerase B and dihydrodipicolinate reductase were used as positive and negative control probes, respectively, to evaluate RNA quality. The results were scored using RNAscope® Spot Studio Software (ACD) as follows: “0,” no staining or <1 dot per 10 cells; “1,” 1-3 dots per cell; “2,” 4-9 dots per cell or very few clusters; “3,” 10-15 dots per cell or <10% dots in clusters; and “4,” >15 dots per cell or >10% dots in clusters. When the score of the positive control probes was ≥2, the tissue section was defined as qualified.

### Immunohistochemical staining

FFPE tissue slides were deparaffinized with xylene and rehydrated through a graded alcohol series. Next, endogenous peroxidase activity was blocked using hydrogen peroxide. After antigen retrieval, FFPE slides were incubated with an anti-PDGFRA antibody (dilution 1:250; Santa Cruz Biotechnology, Santa Cruz, CA, USA) overnight in darkness. Horseradish peroxidase (HRP)-conjugated polyclonal rabbit antibody (Dako, Glostrup, Denmark) diluted at 1:400 was used as the secondary antibody. Following antibody incubation, slides were washed twice and then subjected to detection with chromogen aminoethyl carbazole (AEC; ZSGB-BIO, Beijing, China). Specific immunostaining of each sample was scored as 0-3 based on the staining intensity and density by pathologists blinded to the genotype of the samples.

### Plasmid construction and oligonucleotide transfection

Plasmid vectors carrying full-length fragments of *PDGFRA* containing the coding sequence with either the C (pLenti-CMV-PDGFRA(C)-3FLAG-PGK-Puro) or T (pLenti-CMV-PDGFRA(T)-3FLAG-PGK-Puro) allele of rs2228230 were designed and synthesized by Obio Technology (Shanghai, China). The control plasmid pLenti-CMV-3FLAG-PGK-Puro vector was purchased from Obio Technology. Transient transfection of plasmids into HEK293 cells was performed using Lipofectamine 3000 (Invitrogen, Carlsbad, CA, USA).

### Protein and mRNA stability assays

After 48 h of transfection, cells were treated with 5 µg/mL actinomycin D (ActD; APExBIO, Houston, TX, USA) to inhibit RNA synthesis and harvested at 0, 0.5, 1, 2, and 4 h. RNA was extracted and quantified by qRT-PCR to detect mRNA stability. To determine protein stability, cycloheximide (CHX; 20 µg/mL; APExBIO) was added to the culture medium to inhibit protein synthesis for 0, 2, 4, and 8 h, and protein was extracted and detected by western blotting. At 48 h after transfection, cells were starved overnight and exposed to 1 µg/mL MG132 (APExBIO) for 6 h to inhibit protein degradation, and protein was extracted for western blotting.

### Western blotting

Total protein was extracted from cells using RIPA lysis buffer (Beyotime Biotechnology, Shanghai, China) supplemented with protease inhibitors (Roche Diagnostics, Indianapolis, IN, USA). Immunodetection was performed using rabbit anti-PDGFRA, anti-phospho-PDGFRA, anti-AKT, anti-phospho-AKT, anti-ERK1/2, anti-phospho-ERK1/2 (all 1:1000; CST, Danvers, MA, USA) and mouse anti-GAPDH antibodies (1:5000; Abcam, Cambridge, UK) as primary antibodies. HRP-conjugated anti-mouse or anti-rabbit IgG antibodies (1:3000, CST) were used as secondary antibodies. Protein bands were visualized with Amersham ECL Select Western Blotting Detection Reagent (GE Healthcare, Chicago, IL, USA), and band intensities were quantified using ImageJ64.

### *In silico* analysis

To identify the rs2228230 genotype in the TCGA Skin Cutaneous Melanoma (SKCM) dataset, level 2 TCGA SKCM genotype data were extracted from NCI Genomic Data Commons Data Portal (GDC Legacy Archive) with a Birdseed confidence threshold of <0.05 20. RNAseq expression data were extracted as “RNA Seq V2 RSEM” from the level 3 file “rem.genes.normalized_result” using the TCGAbiolinks package in R language. The clinical information was extracted from the GDC portal using the TCGAbiolinks package. The effect of rs2228230:C>T on the mRNA structures and thermodynamics of *PDGFRA* were predicted using the RNAfold Web Server. Codon usage values were obtained from the Codon Usage Database, and the relative synonymous codon usage (RSCU) was calculated as RSCU = S × Nc/Na, where S represents the number of synonymous codons encoding the same amino acid, Nc is the frequency of the codon in the genome, and Na is the relative frequency of the codon for that amino acid 21.

### Statistical analysis

SPSS 21.0 software was used for all statistical analyses. Normally distributed continuous data such as age and Breslow's thickness were evaluated using the two independent sample t-test and described as mean ± SD. Categorical data were described as frequencies and percentages. Genotype frequencies and clinical parameters such as sex, subtype, stage, and metastasis were compared using the χ^2^ test or Fisher's exact test. Relative *PDGFRA* mRNA expression and the H-score of PDGFRA protein were evaluated by the Mann-Whitney U test. Survival curves were established using the Kaplan-Meier method, and the statistical significance of the results was estimated by log-rank tests. Multivariate analysis was carried out using the Cox proportional hazard regression model. Deviations of allele and genotype frequencies were estimated by the Hardy-Weinberg equilibrium. All statistical tests were two-sided, and significance was considered at *P* < 0.05.

## Results

### Regulation of the mRNA secondary structure and expression of *PDGFRA* by the rs2228230 genotype

**Figure 1:**
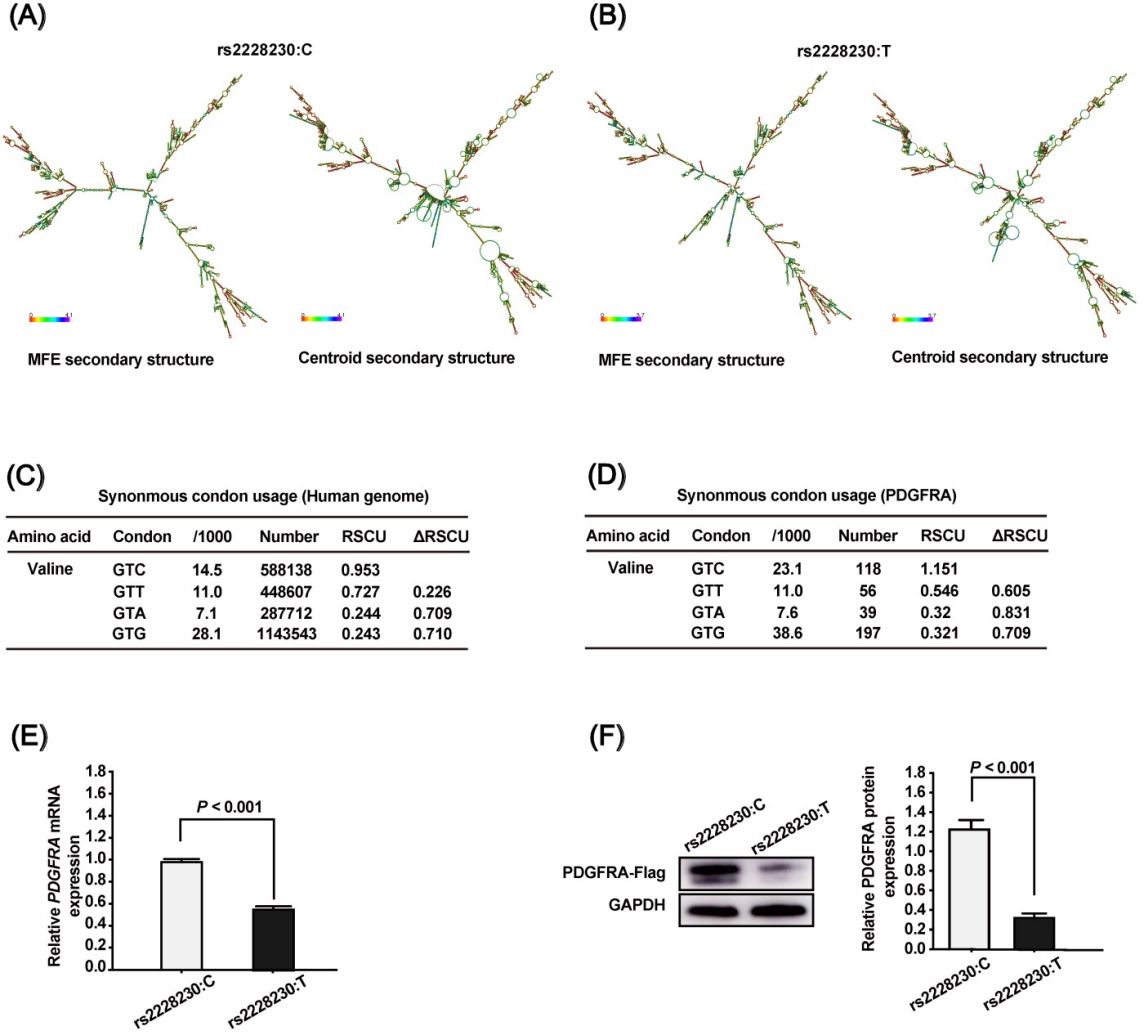
The genotype of rs2228230 affects the mRNA secondary structure and expression of *PDGFRA*. The mRNA secondary structures of full-length *PDGFRA* with the (A) rs2228230:C allele or (B) rs2228230:T allele were predicted using the RNAfold WebServer. Frequencies of the valine codons per thousand and absolute numbers in (C) the genome and (D) *PDGFRA* gene. The relative synonymous codon usage (RSCU) was calculated as RSCU = S × Nc/Na, where S represents the number of synonymous codons encoding the same amino acid, Nc is the frequency of the codon in the genome, and Na is the relative frequency of the codon for that amino acid. *PDGFRA* expression vectors with the rs2228230:C allele or rs2228230:T allele containing a FLAG tag sequence were constructed and transfected into HEK293 cells. After 48 h of transfection, (E) the mRNA expression of *PDGFRA* was evaluated by qRT-PCR, and (F) protein expression was detected by western blotting.

To clarify the association of rs2223230 genotype with *PDGFRA* expression, we conducted a series of *in silico* and *in vitro* analyses. First, the mRNA secondary structure of full-length *PDGFRA* was predicted using the RNAfold WebServer, which showed that the mRNA secondary structure of *PDGFRA* with the rs2228230:C allele (Figure 1A) differed from that with the rs2228230:T allele (Figure 1B). The minimum free energy (MFE) values of *PDGFRA* with the rs2228230:C allele and rs2228230:T allele were predicted to be -1392.95 kcal/mol and -1478.56 kcal/mol, respectively, indicating that the stability of *PDGFRA* mRNA with the rs2228230:T allele was lower than that with the rs2228230:C allele. Second, we extracted the codon usage values and calculated the RSCU values to verify if the rs2228230:T allele could alter the codon usage of valine. We found that the RSCU value of GTC (rs2228230:C) is higher than that of GTT (rs2228230:T) at both the genome (Figure 1C) and *PDGFRA* gene level (Figure 1D). The RSCU value for GTC in *PDGFRA* is greater than 1, which implies a bias for GTC codon usage in the valine synthesis process. These *in silico* analyses indicated that the rs2228230:T allele might reduce *PDGFRA* expression through decreased mRNA stability and lower codon usage.

To validate this hypothesis, we constructed *PDGFRA* expression vectors with the rs2228230:C allele or rs2228230:T allele containing a FLAG tag sequence and transfected them into HEK293 cells. After 48 h of transfection, mRNA was extracted and transcribed into cDNA using a reverse primer that can amplify the FLAG tag to avoid the effect of endogenous *PDGFRA* expression. qRT-PCR showed that the relative expression level of *PDGFRA* mRNA was 1.75-fold lower in cells transfected with the rs2228230:T allele than in cells transfected with the rs2228230:C allele (*P* < 0.001; Figure 1E). Protein expression of PDGFRA was further examined by western blotting after 48 h of transfection, and the results were consistent with those of qRT-PCR; the expression level of PDGFRA was four-fold lower in cells with the rs2228230:T allele (*P* < 0.001; Figure 1F). These results conclusively demonstrated that the genotype of rs2228230 affected the expression of *PDGFRA* at both the mRNA and protein level.

### Influence of the rs2228230 genotype on the mRNA and protein stability of *PDGFRA*

**Figure 2:**
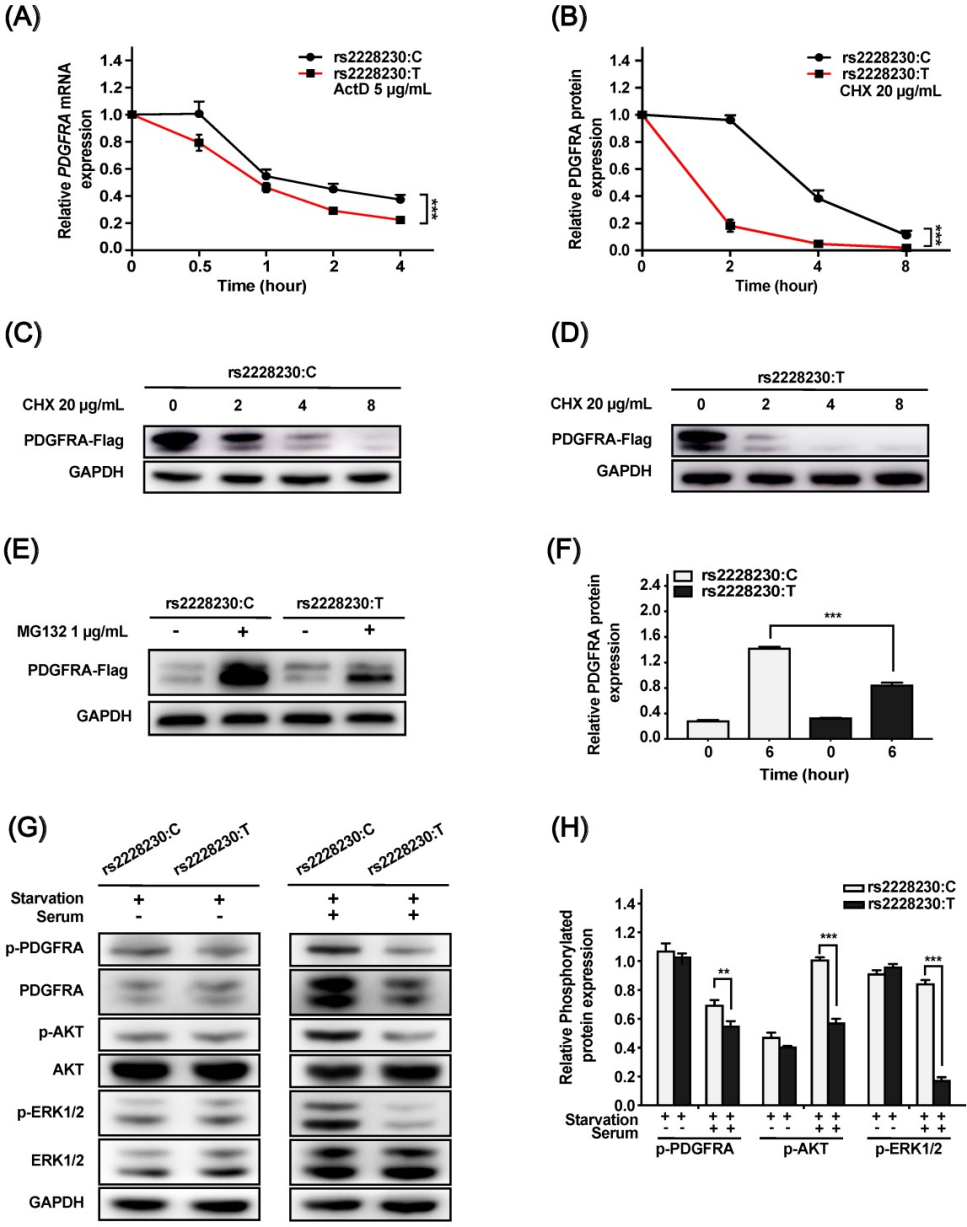
The genotype of rs2228230 alters the stability and signaling activity of PDGFRA. (A) HEK293 cells were transfected with *PDGFRA* expression vectors containing the rs2228230:C allele or rs2228230:T allele. At 48 h after transfection, cells were treated with actinomycin D (ActD) for different time periods to inhibit RNA synthesis, and *PDGFRA* expression was examined by qRT-PCR to evaluate mRNA stability. (B*-*D) Cells were treated with cycloheximide (CHX) to inhibit protein synthesis for different time periods, and protein was extracted for western blotting to detect the proteolytic degradation of PDGFRA. Cells were serum-starved overnight and treated with MG132 for 6 h, and (E) PDGFRA expression was examined by western blotting and (F) quantified to evaluate the protein synthesis rate. Cells were serum-starved overnight, followed by serum stimulation for 60 min. Next, the expression and phosphorylation of PDGFRA, as well as key molecules of MAPK and PI3K/AKT signaling, were (G) detected and (H) quantified. *P < 0.05, **P < 0.01, ***P < 0.001.

We further investigated whether post-transcriptional or post-translational mechanisms were involved in the reduced expression of *PDGFRA* with the rs2228230:T allele. For the mRNA stability assay, ActD was added to the culture medium to inhibit RNA synthesis. As shown in Figure 2A, the mRNA decay rate of *PDGFRA* was significantly higher in cells with the rs2228230:T allele than in cells with the rs2228230:C allele (*P* < 0.001), indicating that *PDGFRA* mRNA stability was reduced by the rs2228230:T allele.

Cells transfected with the rs2228230:C allele or rs2228230:T allele of *PDGFRA* were treated with the protein synthesis inhibitor CHX for various durations. PDGFRA levels decreased with increasing CHX exposure time, and the degradation rate of PDGFRA in cells with the rs2228230:T allele was significantly faster than that in cells with the rs2228230:C allele, indicating that the rs2228230:T allele increased proteolytic degradation (*P* < 0.001; Figure 2B-D).

MG132 is a proteasome inhibitor that can block the activity of the 20S proteasome and the proteolytic activity of the 26S proteasome complex 22. After proteolytic degradation was inhibited, cells with the rs2228230:T allele showed a 43% decrease in PDGFRA translation compared to cells with the rs2228230:C allele (*P* < 0.001; Figure 2E, F), indicating that the rs2228230:T allele also decreased the protein synthesis rate of PDGFRA. Hence, the rs2228230 genotype impacts the mRNA and protein stability of PDGFRA.

### Influence of the rs2228230 genotype on the signaling activity of PDGFRA

As the above investigations demonstrated that the genotype of rs2228230 could influence PDGFRA expression, we hypothesized that it might also affect PDGFRA function. To validate this hypothesis, we analyzed the activation of downstream signaling, including the MAPK and PI3K/AKT pathways. Cells transfected with different genotypes of *PDGFRA* were serum-starved overnight, followed by serum stimulation for 60 min. The basic expression and phosphorylation levels of all molecules were similar after serum starvation (Figure 2G, H); after serum stimulation, the expression levels of total and phosphorylated PDGFRA, phospho-AKT, and phospho-ERK were significantly decreased in cells with the rs2228230:T allele compared to those with the rs2228230:C allele, while the expression levels of total AKT and ERK were not different in the two groups. Thus, the genotype of rs2228230 could influence the signaling activity of PDGFRA.

### Association of the rs2228230 genotype with the mRNA and protein expression of PDGFRA in AM

**Figure 3:**
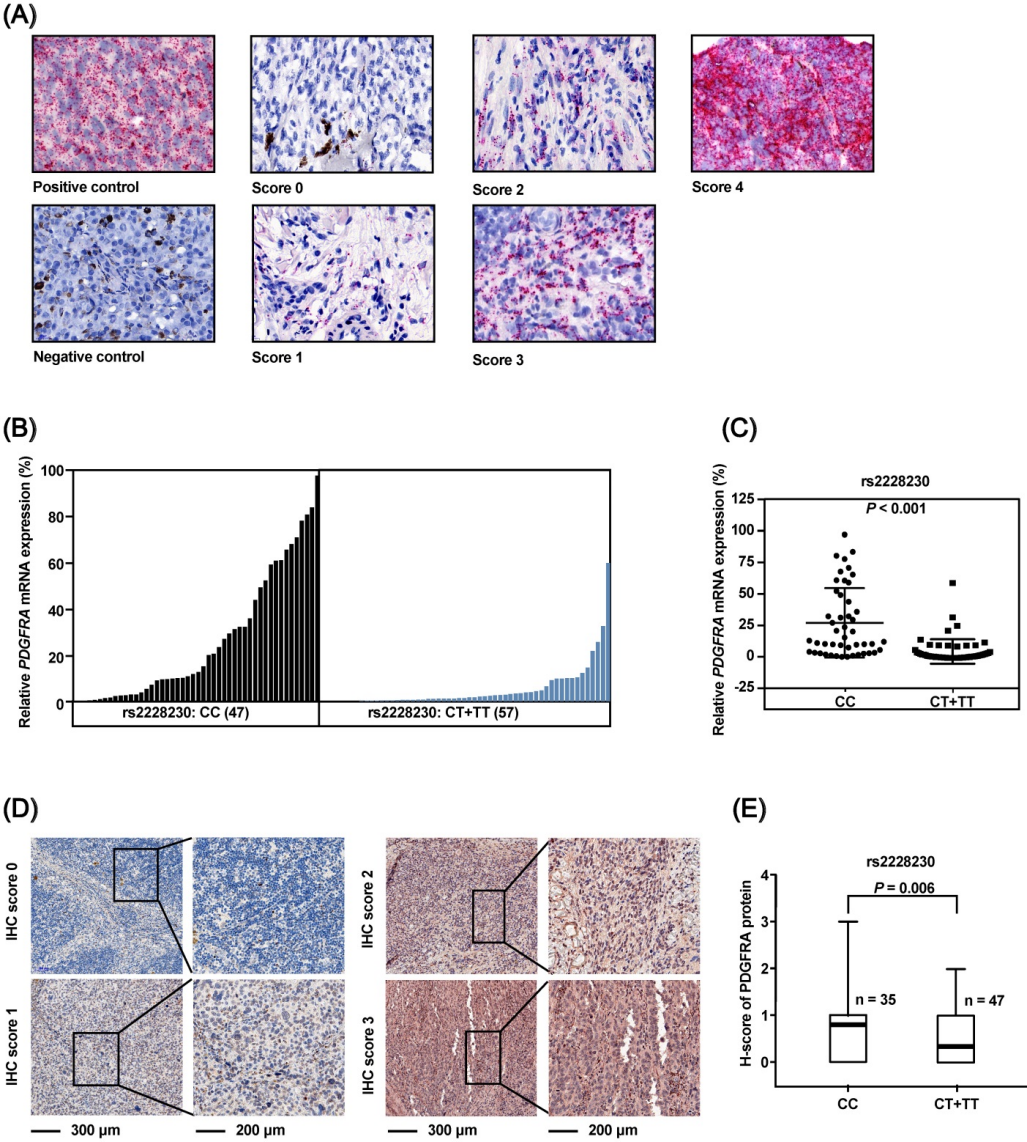
The *PDGFRA* rs2228230 genotype is correlated with PDGFRA mRNA and protein expression level in AM. (A) Representative *in situ* RNAscope hybridization images of *PDGFRA* in FFPE specimens. Each red dot represents a single transcript of *PDGFRA* or the positive control. The relative expression of *PDGFRA* mRNA was normalized to positive control dot counts and is shown in a (B) waterfall plot and a (C) scatter plot. (D) Representative IHC staining images of PDGFRA in AM. Red staining of the cytoplasm and nucleus was considered positive. The staining of each sample was scored as 0-3 according to the staining intensity and density by pathologists blinded to the genotype of the samples. (E) Correlation between PDGFRA expression and rs2228230 genotype. Significance was assessed by the Mann-Whitney U test.

The association of the rs2228230 genotype with expression of *PDGFRA* in AM was validated in FFPE samples, as no commercial AM cell lines were available. The mRNA expression of *PDGFRA* was analyzed by semi-quantitative RNAscope *in situ* hybridization in 104 FFPE AM samples (Figure 3A). The mean expression level of *PDGFRA* mRNA in the CC group was 27.101 (range: 0.000-97.353), while the mean value was 5.068 (range: 0.000-59.542) in the CT+TT group (*P* < 0.001; Figure 3B, C), demonstrating that the *PDGFRA* mRNA expression level was significantly reduced in melanoma tissues with the rs2228230:T genotype.

The association of rs2228230 with PDGFRA protein expression was analyzed by immunohistochemical (IHC) staining in 82 AM tissues. The positive rate of PDGFRA in patients with the rs2228230:T allele (25.5%, 12/47) was significantly lower than in patients with the CC genotype (54.3%, 19/35; *P* = 0.008). IHC staining was scored from 0 to 3 according to staining intensity and density (Figure 3D), and the results showed that the PDGFRA expression level in patients with the CT+TT genotype was significantly lower than that in patients with the CC genotype (*P* = 0.006; Figure 3E). These data indicated that the rs2228230:T allele was associated with decreased expression of *PDGFRA* at both the mRNA and protein level in AM.

### Correlation of rs2228230 genotype with patient characteristics and clinical outcome in AM

The association of the rs2228230 genotype with prognosis in AM was further retrospectively analyzed in a discovery cohort and a replication cohort. The basic clinicopathological characteristics of the patients are summarized in Table S1. The distribution of rs2228230 was in concordance with the Hardy-Weinberg equilibrium in both cohorts, and the minor allele frequency of rs2228230 was as expected in Asians according to the NCBI database of genetic variation dbSNP (Table S2). The correlation of the *PDGFRA* rs2228230 genotype with clinical characteristics of AM was analyzed, and no factor was found to be significantly correlated in either cohort (Table S3).

**Figure 4:**
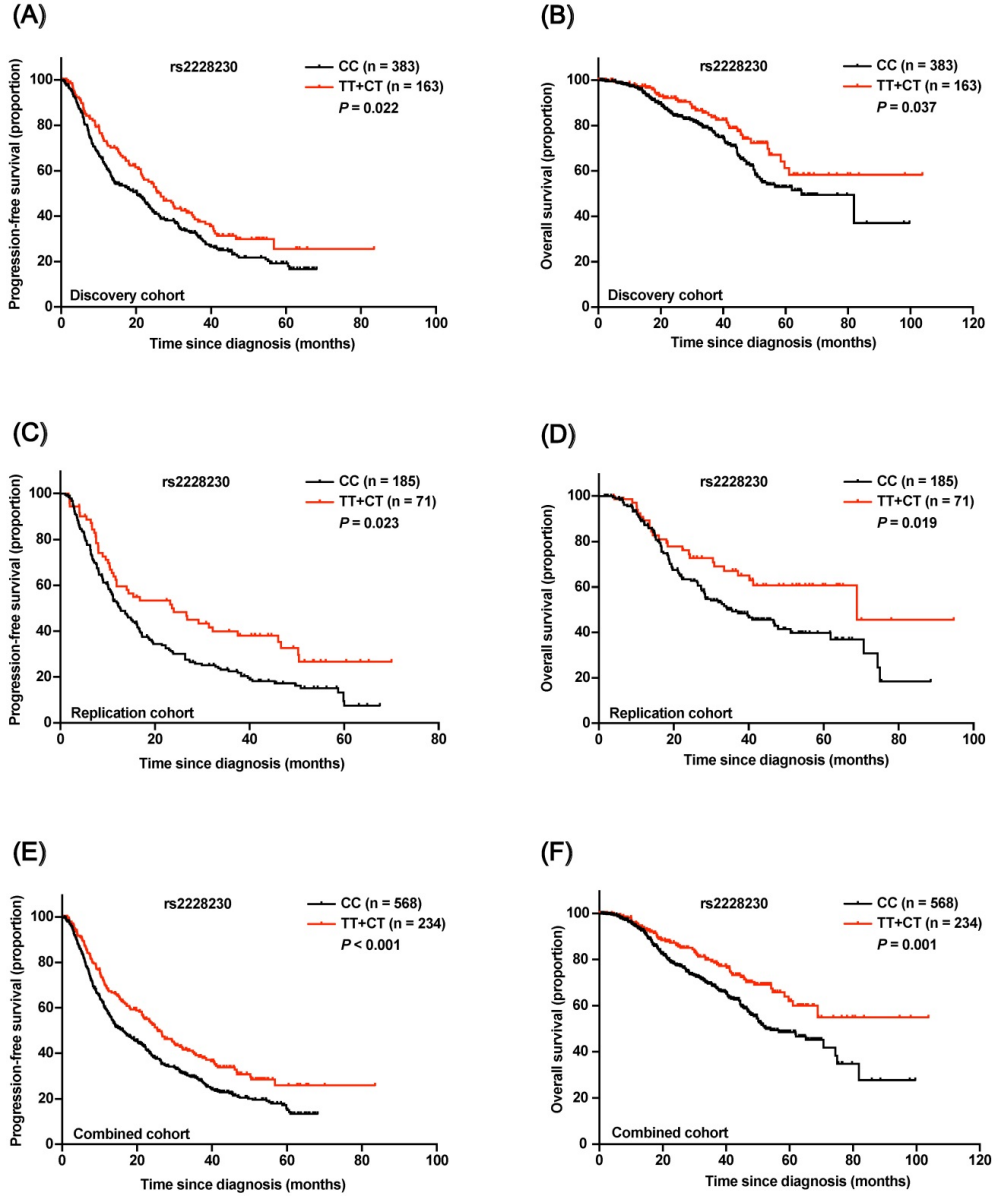
Association of the rs2228230 genotype with prognosis in AM patients. Kaplan-Meier curve of (A) PFS and (B) OS according to the rs2228230 genotype in the discovery cohort. Kaplan-Meier curve of (C) PFS and (D) OS in the replication cohort. Kaplan-Meier plot of (E) PFS and (F) OS according to the rs2228230 genotype in the combined cohort.

In the discovery cohort, Kaplan-Meier survival analysis revealed that both progression-free survival (PFS; *P* = 0.022; Figure 4A) and OS (*P* = 0.037; Figure 4B) of AM patients with the rs2228230:T allele were significantly longer compared to those of patients with the CC genotype. Multivariate Cox regression analysis results are shown in Table 1; the rs2228230:T allele was significantly associated with longer PFS (hazard ratio, HR: 0.696; 95% CI: 0.547-0.884; *P* = 0.003) and OS (HR: 0.630; 95% CI: 0.426-0.932; *P* = 0.021).

In the replication cohort, PFS (HR: 0.622; 95% CI: 0.443-0.873; *P* = 0.006) and OS (HR: 0.614; 95% CI: 0.388-0.972; *P* = 0.037) of patients with the rs2228230:T allele were also longer than those of patients with the CC genotype (Figure 4C, D). The association of the rs2228230 genotype with prognosis was further analyzed in the combined cohort, and the protective effect of the rs2228230:T allele remained significant (Figure 4E, F); AM patients with the rs2228230:T allele had longer PFS (HR: 0.656; 95% CI: 0.540-0.798; *P* < 0.001) and OS (HR: 0.586; 95% CI: 0.436-0.788; *P* < 0.001) compared to patients with the CC genotype.

### Association of the rs2228230 genotype with clinical outcome in CM patients

**Figure 5:**
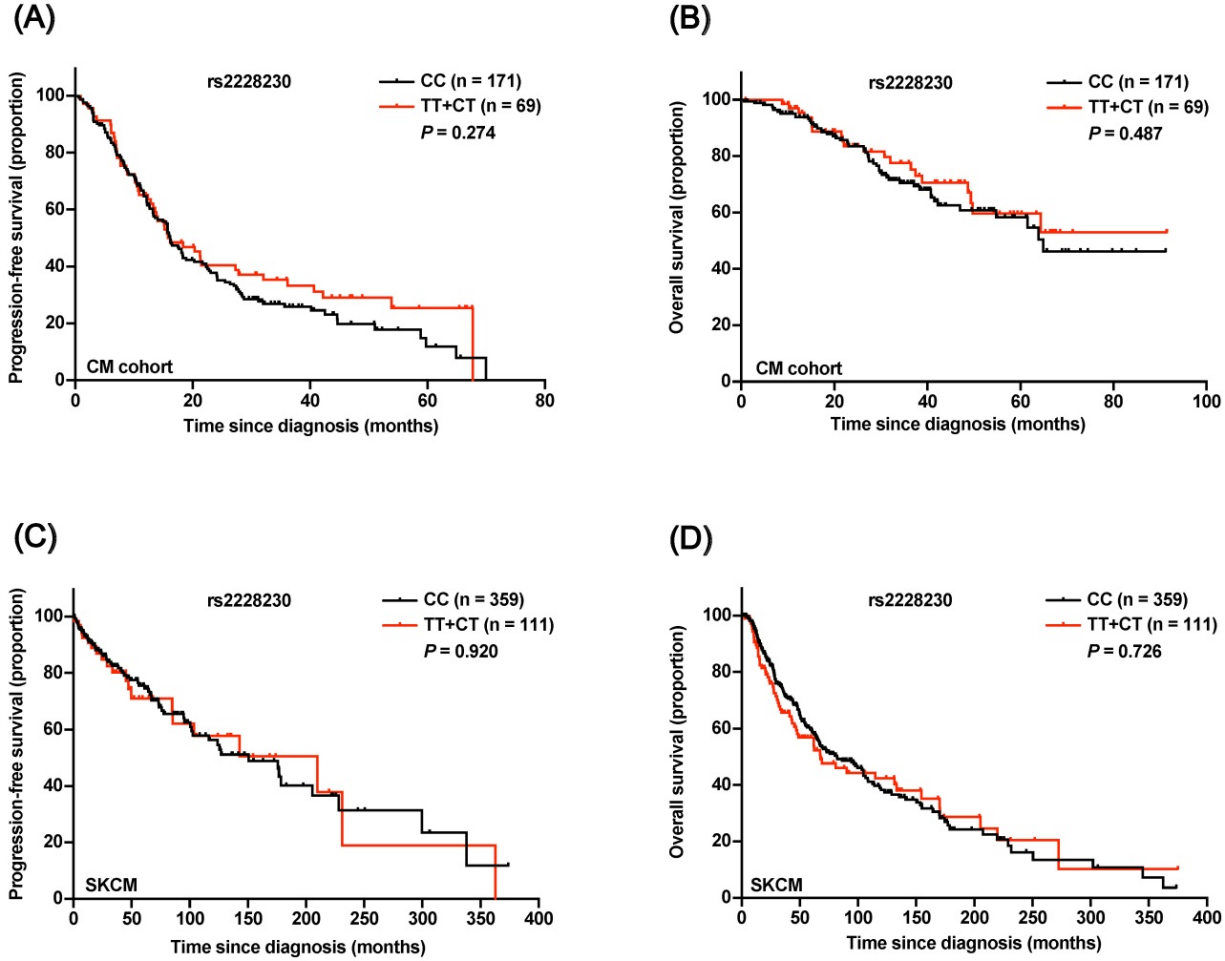
Association of the rs2228230 genotype with prognosis in CM patients. Kaplan-Meier curve of (A) PFS and (B) OS according to the rs2228230 genotype in 240 CM patients. Kaplan-Meier curve of (C) PFS and (D) OS in the TCGA SKCM dataset.

The prognostic significance of rs2228230 was verified in CM. The baseline patient characteristics are summarized in Table S4, and the distribution of rs2228230 was in concordance with the Hardy-Weinberg equilibrium (Table S5). Both Kaplan-Meier survival analysis (Figure 5A, B) and univariate Cox analysis (Table S6) showed that the genotype of rs2228230 was not related to PFS or OS in CM. The association of the rs2228230 genotype with prognosis and *PDGFRA* expression was also analyzed in the TCGA SKCM dataset, in which most of the patients were Caucasian. As in our CM cohort, the genotype of rs2228230 was not related to PFS or OS (Figure 5C, D). Moreover, the mRNA expression of *PDGFRA* was not different between the different genotypes (Figure S2). These results demonstrated that the effect of rs2228230 on *PDGFRA* expression and survival in CM differed according to original site.

## Discussion

This study was conducted to investigate the functional effect of the tag SNP rs2228230 and its clinical significance in AM. rs2228230 is a synonymous SNP (sSNP), which is a specific type of polymorphism that does not change the encoded amino acid. sSNPs were recently demonstrated to alter mRNA and protein expression levels, structures, and functions by different mechanisms 23. For example, the sSNP rs1045642 located in the coding sequence of *ABCB1* can reduce its mRNA expression through biased codon usage, resulting in functional alteration of the substrate 24,25. This *ABCB1* sSNP has been reported to be associated with tacrolimus response in ulcerative colitis 26, imatinib response in chronic myeloid leukemia 27, and paclitaxel sensitivity in different cancer cells 28. Our *in silico* and *in vitro* studies performed to clarify the effect of the rs2228230 genotype on *PDGFRA* expression demonstrated that the mRNA and protein expression decreased in cells transfected with the rs2228230:T allele. mRNA secondary structure analysis revealed that the rs2228230:T allele changed the structure of *PDGFRA* and increased the MFE. A higher MFE implies lower mRNA stability, which might increase mRNA degradation. The *in silico* prediction was verified by an mRNA stability assay. After RNA synthesis was inhibited by ActD, *PDGFRA* mRNA decayed faster in cells with the rs2228230:T allele than in cells with the rs2228230:C allele.

The genetic code is degenerate, but synonymous codons are not used equally; the codon usage bias might alter the rate of translation elongation and therefore affect protein conformation and expression 29-32. According to the codon usage values of four valine codons at the genome and *PDGFRA* gene level, we confirmed that codon usage was biased toward the major allele C, indicating that the translation elongation of PDGFRA would be slower in cells with the rs2228230:T allele. The subsequent experiment demonstrated that after proteolytic degradation was inhibited, the cells with the rs2228230:T allele showed a decreased rate of PDGFRA protein synthesis. Furthermore, the degradation rate of PDGFRA was significantly faster in cells with the rs2228230:T allele after protein synthesis was inhibited, indicating that the rs2228230:T allele also increased proteolytic degradation. Thus, the rs2228230 genotype could affect *PDGFRA* expression by regulating mRNA stability, protein synthesis, and degradation rates. Furthermore, rs2228230:T not only reduced PDGFRA expression but also decreased the activation of the MAPK and PI3K/AKT pathways.

Other molecular mechanisms by which sSNPs within coding sequences can affect protein expression and function have been reported. First, coding-region sSNPs can affect splicing accuracy or efficiency by altering the binding of splicing regulatory proteins to ESS or ESE in exons 33,34. Compared to the major allele, the minor T allele of rs2228230 reduces a serine- and arginine-rich splicing factor 2 (SRSF2)-binding site and increases a serine- and arginine-rich splicing factor 6 (SRSF6)-binding site 35,36. Both SRSF2 and SRSF6 are arginine-rich proteins; however, SRSF2 is located only in the nucleus, whereas SRSF6 can shuttle between the nucleus and cytoplasm 37, indicating that besides altering mRNA stability, rs2228230 might also affect mRNA splicing. Second, sSNPs in coding sequences can also affect microRNA-binding sites. For example, the minor allele of rs10065172 reduces the binding capacity to miR-196 and therefore dysregulates the expression of *IRGM* in Crohn's disease 38. Third, tRNA concentrations vary among organs, and differences in tRNA levels are a major determinant of translation elongation speed 39. It has been demonstrated that the sSNP T2562G within *CFTR* alters the local translation speed and therefore changes CFTR stability and function by introducing a low-abundance tRNA in a bronchial epithelial tissue-specific manner 40. Moreover, sSNPs might influence the protein folding process and result in misfolded protein and decreased expression thereof 41.

In this study, we found that the minor allele T of rs2228230 was associated with reduced mRNA and protein expression of *PDGFRA* in AM samples, and the following study in two independent cohorts demonstrated that AM patients with the rs2228230:T allele had longer PFS and OS compared to patients with the CC genotype, indicating that it may be used as a predictor of improved outcome. However, the rs2228230 genotype was not found to be correlated with survival in our CM cohort or the TCGA SKCM dataset. These results indicated that the prognostic value of rs2228230:T varies based on original site, which might be because UV radiation-induced genetic aberration is more extensive in CM, and other molecular variations could affect *PDGFRA* expression and signaling activation. Patterns of linkage disequilibrium differ across races/ethnicities 42,43, and the expression of *PDGFRA* might be epigenetically regulated 44. Therefore, the prognostic value of rs2228230 in other populations must be further validated in prospective studies.

In conclusion, our study showed that the minor T allele of rs2228230 can reduce the expression and function of PDGFRA by altering the stability and synthesis of its mRNA and protein, and that it is associated with better survival in AM patients. These results highlight the potential of the rs2228230 genotype to serve as a prognostic marker in AM.

## Supplementary Material

Supplementary figures and tables.

## Figures and Tables

**Table 1:** Association of *PDGFRA* rs2228230 genotype with PFS and OS in AM

			PFS					OS			
	Clinical character	Group	Univariate HR (95% CI)	*P* value	Multivariate HR (95% CI)	*P* value		Univariate HR (95% CI)	*P* value	Multivariate HR (95% CI)	*P* value
**Discovery cohort**	Sex	Male vs. Female	1.190 (0.965-1.468)	0.103				1.382 (0.988-1.934)	0.059		
	Age	≥60 vs. <60	0.941 (0.753-1.175)	0.590				0.785 (0.543-1.133)	0.195		
	Thickness	≥4 vs. <4	0.993 (0.802-1.229)	0.948				1.239 (0.866-1.734)	0.211		
	TNM	III/IV vs. I/II	2.854 (2.296-3.549)	<0.001*	2.926 (2.352-3.640)	<0.001*		3.532 (2.443-5.017)	<0.001*	3.581 (2.477-5.178)	<0.001*
	rs2228230	CT/TT vs. CC	0.756 (0.595-0.960)	0.022*	0.696 (0.547-0.884)	0.003*		0.660 (0.447-0.976)	0.037*	0.630 (0.426-0.932)	0.021*
	*CKIT*	Mut vs. WT	1.023 (0.707-1.479)	0.906				0.868 (0.469-1.608)	0.654		
	*BRAF*	Mut vs. WT	1.148 (0.887-1.486)	0.293				0.856 (0.547-1.340)	0.497		
	*NRAS*	Mut vs. WT	1.281 (0.956-1.716)	0.097				1.319 (0.829-2.099)	0.236		
	*PDGFRA*	Mut vs. WT	0.892 (0.369-2.158)	0.801				1.265 (0.403-3.973)	0.687		
**Replication cohort**	Sex	Male vs. Female	1.004 (0.753-1.338)	0.979				1.093 (0.754-1.586)	0.638		
	Age	≥60 vs. <60	0.931 (0.690-1.256)	0.639				1.022 (0.694-1.505)	0.913		
	Thickness	≥4 vs. <4	0.844 (0.621-1.146)	0.276				1.066 (0.710-1.600)	0.757		
	TNM	III/IV vs. I/II	1.762 (1.268-2.448)	0.001*	1.883 (1.353-2.622)	<0.001*		2.172 (1.361-3.467)	0.001*	2.340 (1.463-3.743)	<0.001*
	rs2228230	CT/TT vs. CC	0.625 (0.447-0.874)	0.006*	0.622 (0.443-0.873)	0.006*		0.593 (0.377-0.932)	0.023*	0.614 (0.388-0.972)	0.037*
	*CKIT*	Mut vs. WT	1.068 (0.657-1.737)	0.790				1.140 (0.593-2.190)	0.694		
	*BRAF*	Mut vs. WT	1.114 (0.809-1.618)	0.446				1.092 (0.680-1.756)	0.715		
	*NRAS*	Mut vs. WT	1.806 (1.205-2.706)	0.004*	1.749 (1.162-2.634)	0.007*		2.541 (1.583-4.080)	<0.001*	2.432 (1.504-3.935)	<0.001*
	*PDGFRA*	Mut vs. WT	0.566 (0.140-2.287)	0.424				0.455 (0.063-3.261)	0.433		
**Combined cohort**	Sex	Male vs. Female	1.105 (0.933-1.307)	0.247				1.193 (0.932-1.528)	0.162		
	Age	≥60 vs. <60	0.935 (0.782-1.117)	0.458				0.892 (0.685-1.163)	0.399		
	Thickness	≥4 vs. <4	1.004 (0.849-1.188)	0.960				1.357 (1.058-1.741)	0.016*	1.184 (0.921-1.521)	0.187
	TNM	III/IV vs. I/II	2.465 (2.061-2.949)	<0.001*	2.536 (2.118-3.036)	<0.001*		3.238 (2.429-4.316)	<0.001*	3.232 (2.420-4.315)	<0.001*
	rs2228230	CT/TT vs. CC	0.707 (0.582-0.859)	<0.001*	0.656 (0.540-0.798)	<0.001*		0.615 (0.458-0.827)	0.001*	0.586 (0.436-0.788)	<0.001*
	*CKIT*	Mut vs. WT	1.042 (0.777-1.398)	0.781				0.998 (0.639-1.561)	0.995		
	*BRAF*	Mut vs. WT	1.154 (0.938-1.419)	0.175				0.948 (0.685-1.313)	0.748		
	*NRAS*	Mut vs. WT	1.426 (1.126-1.805)	0.003*	1.346 (1.063-1.705)	0.014*		1.676 (1.206-2.328)	0.002*	1.557 (1.119-2.167)	0.009
	*PDGFRA*	Mut vs. WT	0.783 (0.371-1.650)	0.520				0.847 (0.315-2.276)	0.742		

*, *P* < 0.05. AM, acral melanoma; PFS, progression-free survival; OS, overall survival; HR, hazard ratio; TNM, tumor-node-metastasis stage; Mut, mutation; WT, wild type.

## Abbreviations

ActD: actinomycin D
AM: acral melanoma
CHX: cycloheximide
CM: cutaneous melanoma
ESE: exonic splicing enhancer
ESS: exonic splicing silencer
FFPE: formalin-fixed, paraffin-embedded
GWAS: genome-wide association study
HR: hazard ratio
HRP: horseradish peroxidase
IHC: immunohistochemical
MFE: minimum free energy
OS: overall survival
PDGF: platelet-derived growth factor
PDGFRA: PDGF receptor alpha
PFS: progression-free survival
qRT-PCR: quantitative reverse transcription polymerase chain reaction
RSCU: relative synonymous codon usage
SKCM: Skin Cutaneous Melanoma
SNP: single-nucleotide polymorphism
SRSF2: serine- and arginine-rich splicing factor 2
SRSF6: serine- and arginine-rich splicing factor 6
sSNP: synonymous SNP
