# Supplementary material for: A Functional Synonymous Variant in PDGFRA Is Associated with Better Survival in Acral Melanoma
Source: J Cancer. 2020 Mar 4;11(10):2945–56. doi: 10.7150/jca.43010 (PMC7086247; doi:10.7150/jca.43010)
Supplement: Supplementary file 1 — Supplementary figures and tables. [file jcav11p2945s1.pdf]

Table S1. Baseline patient characteristics of the AM discovery and replication cohorts

Cohort	Discovery (N=546)		Replication (N=256)		Combined (N=802)	
	N	%	N	%	N	%
Sex						
Female	267	48.9	146	57.0	413	51.5
Male	279	51.1	110	43.0	389	48.5
Age	53.83 ± 13.08 (8 - 92)		54.44 ± 11.75 (15 - 78)		54.02 ± 12.66 (8 - 92)	
<60	359	65.8	168	65.6	525	65.7
≥60	187	34.2	88	34.4	275	34.3
Thickness	3.85 ± 2.98 (0 - 19.5)		5.93 ± 3.28 (0 - 30)		4.54 ± 3.33 (0 - 30)	
<4	317	58.1	74	28.9	391	48.8
≥4	229	41.9	182	71.1	411	51.2
TNM						
I	43	7.9	4	1.6	47	5.9
II	230	42.1	73	28.5	303	37.8
III	213	39.0	126	49.2	339	42.3
IV	60	11.0	53	20.7	113	14.1
CKIT						
Wild type	498	91.2	233	91.0	731	91.1
Mutation	48	8.8	23	9.0	71	8.9
BRAF						
Wild type	437	80.0	206	80.5	643	80.2
Mutation	109	20.0	50	19.5	159	19.8
NRAS						
Wild type	471	86.3	219	85.5	690	86.0
Mutation	75	13.7	37	14.5	112	14.0
PDGFRA						
Wild type	537	98.4	253	98.8	790	98.5
Mutation	9	1.6	3	1.2	12	1.5

AM, acral melanoma; TNM, tumor-node-metastasis stage.

Table S2. Genotype and allele frequency of *PDGFRA* rs2228230 and Hardy–Weinberg equilibrium test in the AM cohorts

**rs2228230:C>T**	**Discovery cohort**						**Replication cohort**					
	**Genotype**			**Allele**		***P* value**[a]	**Genotype**			**Allele**		***P* value**[a]
	**CC**	**CT**	**TT**	**C**	**T**		**CC**	**CT**	**TT**	**C**	**T**	
Count	383	147	16	913	179	0.969	186	61	9	433	79	0.636
Frequency	70.1%	26.9%	2.9%	83.6%	16.4%		72.7%	23.8%	3.5%	84.6%	15.4%	

AM, acral melanoma.

[a] The *P* value of the Hardy–Weinberg equilibrium test was analyzed by the χ^2 test.

Table S3. Correlation of *PDGFRA* rs2228230 genotype with clinical characteristics of AM

		Discovery cohort			Replication cohort			Combined cohort		
**Clinical character**	**Group**	**CC**	**CT+TT**	***P* value[a]**	**CC**	**CT+TT**	***P* value[a]**	**CC**	**CT+TT**	***P* value[a]**
Sex	Female	185 (48.3%)	82 (50.3%)	0.668	102 (55.1%)	44 (62.0%)	0.323	287 (50.5%)	126 (53.8%)	0.393
	Male	198 (51.7%)	81 (49.7%)		83 (44.9%)	27 (38.0%)		281 (49.5%)	108 (46.2%)	
Age	<60	257 (71.0%)	102 (62.6%)	0.308	123 (66.5%)	45 (63.4%)	0.639	380 (66.9%)	147 (62.8%)	0.268
	≥60	126 (32.9%)	61 (37.4%)		62 (33.5%)	26 (36.6%)		188 (33.1%)	87 (37.2%)	
Thickness (mm)	<4	218 (56.9%)	99 (60.7%)	0.546	58 (31.4%)	16 (22.5%)	0.164	276 (48.6%)	115 (49.1%)	0.887
	≥4	165 (43.1%)	64 (39.3%)		127 (68.6%)	55 (77.5%)		292 (51.4%)	119 (50.9%)	
TNM stages	I	29 (7.6%)	14 (8.6%)	0.436	2 (1.1%)	2 (2.8%)	0.597	31 (5.5%)	16 (6.8%)	0.358
	II	167 (43.6%)	63 (38.7%)		56 (30.3%)	17 (23.9%)		223 (39.3%)	80 (34.2%)	
	III	142 (37.1%)	71 (43.6%)		89 (48.1%)	37 (52.1%)		231 (40.7%)	108 (46.2%)	
	IV	45 (11.7%)	15 (9.2%)		38 (20.5%)	15 (21.1%)		83 (14.6%)	30 (12.8%)	
Metastasis	No	196 (51.2%)	77 (47.2%)	0.400	58 (31.4%)	19 (26.8%)	0.473	254 (44.7%)	96 (41.0%)	0.338
	Yes	187 (48.8%)	86 (52.8%)		127 (68.6%)	52 (73.2%)		314 (55.3%)	138 (59.0%)	
CKIT	WT	353 (92.2%)	145 (89.0%)	0.225	169 (91.4%)	64 (90.1%)	0.762	522 (91.9%)	209 (89.3%)	0.241
	Mut	30 (7.8%)	18 (11.0%)		16 (8.6%)	7 (9.9%)		46 (8.1%)	25 (10.7%)	
BRAF	WT	307 (80.2%)	130 (79.8%)	0.914	149 (80.5%)	57 (80.3%)	0.963	456 (80.3%)	187 (79.9%)	0.906
	Mut	76 (19.8%)	33 (20.2%)		36 (19.5%)	14 (19.7%)		112 (19.7%)	47 (20.1%)	
NRAS	WT	332 (86.7%)	139 (85.3%)	0.662	154 (83.2%)	65 (91.5%)	0.091	486 (85.6%)	204 (87.2%)	0.548
	Mut	51 (13.3%)	24 (14.7%)		31 (16.8%)	6 (8.5%)		82 (14.4%)	30 (12.8%)	
PDGFRA	WT	377 (98.4%)	160 (98.2%)	0.762	183 (98.9%)	70 (98.6%)	0.828	560 (98.6%)	230 (98.3%)	0.750
	Mut	6 (1.6%)	3 (1.8%)		2 (1.1%)	1 (1.4%)		8 (1.4%)	4 (1.7%)	

AM, acral melanoma; TNM, tumor-node-metastasis stage; Mut, mutation; WT, wild type.

[a] For evaluation of clinical parameters and genotype frequencies, the χ^2 test or Fisher's exact test was used.

Table S4. Baseline patient characteristics of the CM cohort

	N	%
Sex		
Female	128	53.3
Male	112	46.7
Age	46.21±16.65 (8-87)	
<60	193	80.4
≥60	47	19.6
Thickness	4.36±3.15 (0-18.5)	
<4	130	54.2
≥4	110	45.8
TNM		
I	10	4.2
II	79	32.9
III	114	47.5
IV	37	15.4
CKIT		
Wild type	234	97.5
Mutation	6	2.5
BRAF		
Wild type	125	52.1
Mutation	115	47.9
NRAS		
Wild type	229	95.4
Mutation	11	4.6
PDGFRA		
Wild type	238	99.2
Mutation	2	0.8

CM, cutaneous melanoma; TNM, tumor-node-metastasis stage.

Table S5. Genotype and allele frequency of *PDGFRA* rs2228230 and Hardy–Weinberg equilibrium test in the CM cohort

rs2228230:C>T	Genotype			Allele		*P* value[a]
	CC	CT	TT	C	T	
Count	171	58	11	400	80	0.332
Frequency	71.3%	24.2%	4.6%	83.3%	16.7%	

[a] The *P* value of the Hardy–Weinberg equilibrium test was analyzed by the χ^2 test.

Table S6. Association of *PDGFRA* rs2228230 genotype with PFS and OS in CM

		PFS				OS			
**Clinical character**	**Group**	**Univariate HR (95% CI)**	***P* value**	**Multivariate HR (95% CI)**	***P* value**	**Univariate HR (95% CI)**	***P* value**	**Multivariate HR (95% CI)**	***P* value**
Sex	Male vs. Female	0.836 (0.617-1.132)	0.247			0.849 (0.534-1.349)	0.488		
Age	≥60 vs. <60	0.869 (0.588-1.283)	0.639			0.879 (0.482-1.602)	0.673		
Thickness	≥4 vs. <4	1.004 (0.743-1.356)	0.980			0.732 (0.454-1.179)	0.199		
TNM	III/IV vs. I/II	2.554 (1.818-3.590)	0.001*	2.554 (1.818-3.590)	<0.001*	2.523 (1.463-4.350)	0.001*	2.523 (1.463-4.350)	0.001*
rs2228230	CT/TT vs. CC	0.829 (0.593-1.160)	0.274			0.833 (0.496-1.397)	0.487		
CKIT	Mut vs. WT	1.113 (0.412-3.001)	0.833			1.453 (0.456-4.624)	0.527		
BRAF	Mut vs. WT	1.347 (0.998-1.818)	0.051			1.035 (0.652-1.644)	0.883		
NRAS	Mut vs. WT	0.810 (0.380-1.727)	0.585			1.239 (0.452-3.398)	0.678		
PDGFRA	Mut vs. WT	3.620 (0.885-14.804)	0.073			2.794 (0.385-20.269)	0.310		

PFS, progression-free survival; OS, overall survival; CM, cutaneous melanoma; HR, hazard ratio; TNM, tumor-node-metastasis stage; Mut, mutation; WT, wild type.

Supplementary Figure Legends

Figure S1. Linkage disequilibrium (LD) plot of tag SNPs of *PDGFRA*. Candidate tag SNPs of *PDGFRA* were selected based on the public database dbSNP according to the following criteria: SNPs with minor-allele frequencies ≥ 0.05 in the East Asian population; tag SNPs in the Asian population from dbSNP with $r^2 > 0.8$ determined by SNPinfo Web Server. The colors indicate the strength of pairwise LD according to r^2 metrics. The color of each SNP reflects its chromosome position: red represents exon variant, black represents intron variant, blue represents 3′ untranslated region (UTR) variant, and green represents 5′UTR variant. Tag SNPs are marked with red dots.

Figure S2. Association of the rs2228230 genotype with *PDGFRA* expression in the TCGA SKCM dataset. The relative expression of *PDGFRA* mRNA was normalized using the TCGAbiolinks package in R language and is shown in a scatter plot.

Figure S1

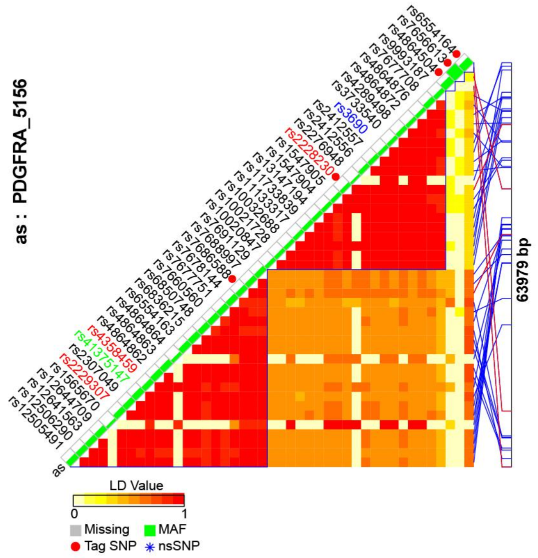

Figure S2

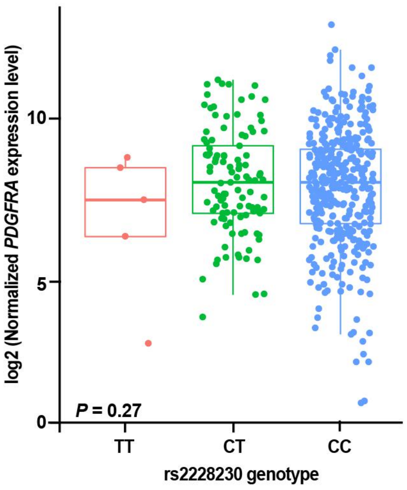
log2 (Normalized PDGFRA expression level)
10
5
0
P = 0.27
TT
CT
CC
rs2228230 genotype
